# Impact of Procalcitonin-Guided Antibiotic Management on Antibiotic Exposure and Outcomes: Real-world Evidence

**DOI:** 10.1093/ofid/ofx213

**Published:** 2017-10-03

**Authors:** Michael R Broyles

**Affiliations:** Department of Clinical Pharmacy and Laboratory Services, Five Rivers Medical Center, Pocahontas, Arkansas

**Keywords:** adverse drug events (ADEs), antibiotic stewardship, procalcitonin, *C. difficile* (CDI)

## Abstract

**Background:**

Delayed pathogen identification and nonspecific clinical findings make definitive decisions regarding antibiotics challenging. The stimuli of bacterial toxins and inflammation make procalcitonin (PCT) unique in its ability to differentiate bacterial infection from other causes of inflammation, and thus it is useful for antibiotic management. The objective of our study was to evaluate the impact of a PCT algorithm (PCT-A) on current practice.

**Methods:**

A single-center, retrospective cohort study was conducted to evaluate the impact of adding PCT-A to stewardship practices. Data from 4 years prior to and after PCT-A implementation were compared in critical and acute care patients of all ages receiving parenteral antibiotics for a DRG coded for infection. A baseline PCT was obtained on admission in patients with suspected bacterial infection. Serial PCT measurements were repeated daily to evaluate effectiveness of therapy. Outcomes of interest were antibiotic exposure, hospital mortality, 30-day readmission, *Clostridium difficile* infection (CDI), and adverse drug events during hospitalization.

**Results:**

A total of 985 patients (pre-PCT-A group) were compared with 1167 patients (post-PCT-A group). Antimicrobial stewardship alone (pre-PCT-A) resulted in a median days of therapy (DOT) of 17 (interquartile range [IQR], 8.5–22.5) vs 9.0 (IQR, 6.5–12) in the post-PCT-A group (*P* < .0001). Secondary outcomes were also significantly reduced in the post-PCT-A group.

**Conclusion:**

The addition of PCT in a facility with an established stewardship program resulted in a significant reduction in antibiotic exposure and adverse outcomes. PCT may improve antibiotic management when diagnostic clarity and resolution of infection are lacking.

Antimicrobial resistance (AMR) is a growing problem, threatening the health of patients in every hospital and community [1]. Antibiotics are the most commonly prescribed medication in the United States and are considered the most significant contributor to both AMR and *Clostridium difficile* infection (CDI) [2].

When antibiotics are properly prescribed to treat bacterial infection, they are effective and should be administered without delay. Increasingly, however, guidance intended to shorten time to initial therapy often pressures clinicians to prescribe broad-spectrum antibiotics within a relatively short window of time [3]. Delay or lack of pathogen identification and nonspecific clinical or radiographic findings often leave clinicians with insufficient evidence to make definitive decisions regarding the need for antibiotics. This may explain the Centers for Disease Control and Prevention’s (CDC’s) findings that nearly half of all antibiotic prescriptions lack proper dosing or indication, rendering them ineffective to treat bacterial infection, contributing to the development of resistant pathogens [2].

Improved antibiotic prescribing practices are the most essential action for preventing the development of resistant bacteria and CDI [2, 4]. Several studies show that procalcitonin (PCT) algorithms used to guide initiation and discontinuation of antibiotics decrease antibiotic exposure without increasing adverse clinical outcomes [5–16]. PCT is a biomarker produced by a host response to bacterial infection and is regulated by microbial toxins and inflammatory cytokines. Initial PCT production occurs at the site of infection and then throughout the body if the infection spreads. The approximate half-life of PCT is 24 hours, and a daily decline is seen in well-controlled infections [17, 18]. Viral pathogens do not elicit the same PCT response, which is attenuated by interferon production [18]. Therefore, PCT has the unique ability to denote the presence of bacterial infection and provide objective information regarding the necessity and optimal duration of antibiotic therapy [17, 18].

The objective of this study was to evaluate the impact of a PCT algorithm (PCT-A) to guide antibiotic management in a real-world setting of patients presenting to a rural community hospital with suspected infection. We hypothesized that the addition of PCT to an established antibiotic stewardship program would thwart unwarranted antibiotics and decrease adverse outcomes. The primary outcome was median days of therapy (DOT), and the secondary outcomes included hospital mortality, 30-day readmission, CDI during hospitalization, and antimicrobial adverse drug events (ADEs) during hospitalization.

## METHODS

### Patients and Setting

This single-center, pre-post, retrospective cohort study was conducted at a 50-bed rural community hospital for the purpose of evaluating the impact of adding a PCT-A to existing antimicrobial stewardship practices. Prior to the PCT-A, the institution had antimicrobial stewardship processes consistent with current recommendations from the American Society of Health System Pharmacists and the Infectious Diseases Society of America (IDSA) [19, 20]. This included pharmacist-coordinated interventions to assess antibiotic use, selection of appropriate agents, dosing, duration of therapy, and route of administration in patients with suspected infection and prescription of antibiotics.

The institutional review board (IRB) approved review of data between the years of 2006 and 2014, and informed consent was waived to evaluate patient records with diagnostic-related group (DRG) codes consistent with infection (supplementary data). Data from 4 years prior to (pre-PCT-A) and after PCT-A implementation (post-PCT-A) were compared in critical and acute care patients. Four-year blocks were chosen prior to data collection, with the intent of reaching 1000 patient records in each cohort. Other time blocks were not explored or analyzed.

All patients who received parenteral antibiotics for infection, inclusive of all ages and immuncompetency status, were eligible for inclusion. Patients who received antibiotics for surgical prophylaxis or those who were transferred to facilities with higher acuity were excluded from the analysis. Data from 2010, the time when hospital staff was oriented to the PCT-A, were not collected or analyzed as this was considered a washout period.

### Intervention

**Figure 1.**
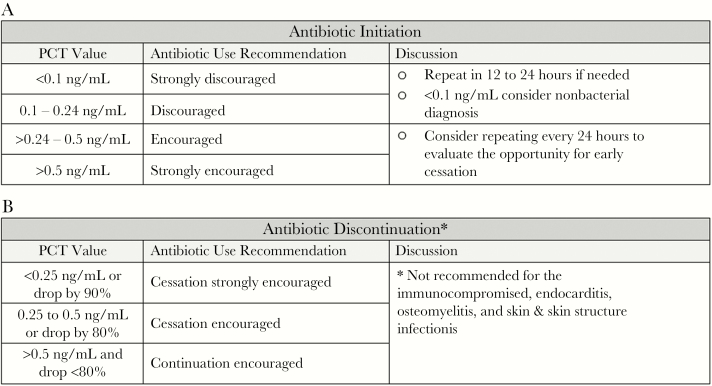
**(A)** Procalcitonin algorithm (PCT-A) for antibiotic initiation (applied in the clinical judgment and other laboratory results). **(B)** PCT-A for antibiotic discontinuation (applied in the context of clinical judgment and other laboratory results). Abbreviation: PCT, procalcitonin.

VIDAS B.R.A.H.M.S. procalcitonin (PCT; bioMerieux, Raleigh, NC) was added as an in-house test with a 1-hour turnaround time in 2010. The PCT-A was derived from previously validated algorithms and added to existing stewardship practices for all patients with suspected or confirmed infections (Figure 1, A and B) [5–16].

Prior to implementation, the PCT-A and suggested treatment guidance were reviewed and approved by the medical staff and pharmacy. To ensure the baseline PCT was not overlooked in the setting of a busy admission, PCT was a prechecked field on the admission orderset for suspected infection. Although providers could override the PCT order, the pharmacists had the ability to order PCT if deemed appropriate for antimicrobial management. Such cases were discussed with providers.

**Figure 2.**
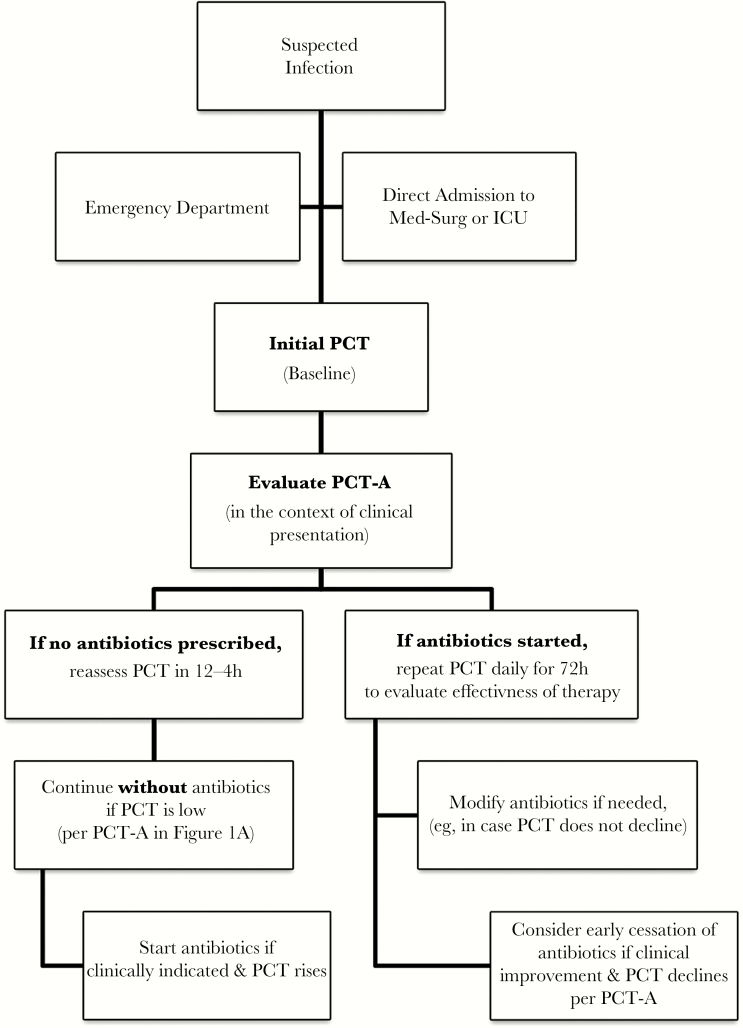
Procalcitonin decision tree for providers and pharmacists. Abbreviations: ICU, intensive care unit; PCT, procalcitonin.

The pharmacists assumed oversight and provided progressive education sessions including presentations, PCT-A pocket cards, and case review for all clinicians prior to and during implementation. The PCT-A was electronically built into the laboratory results and was easily accessible by hovering over the result. PCT-A initiation and follow-up were also overseen by the pharmacists to ensure that PCT was ordered for appropriate patients, results were evaluated, and therapeutic modifications were reviewed with clinicians at the time of result. During the time of admission, clinical management was initiated by ED physicians, and until 2012, management of in-patients was overseen by the hospitalist service or the admitting primary care physician. Starting in 2012, the hospitalist service directed care of all admitted patients. A baseline PCT measurement was obtained upon admission to assess the presence and severity of a bacterial infection. Antibiotics were not recommended in clinically stable patients with low PCT values and low likelihood of bacterial infection. If antibiotics were withheld, PCT was repeated within 24 hours. For patients prescribed antibiotics, serial PCT measurements were repeated daily for the first 72 hours to evaluate the effectiveness of therapy (Figure 2). Therapy modifications were made after the first 24 hours, if required, based on clinical presentation and changes in PCT. Cessation of antibiotics was suggested when clinical disposition had improved and PCT had decreased by more than 80% to 90% or had reached a value of 0.25–0.5 ng/mL.

### Study Data, Outcomes, and Statistics

Cohort characteristics including age, gender, DRG, severity of illness, and antimicrobials were collected electronically through MedHost hospital information system (Franklin, TN). Case mix index (CMI) was used as a surrogate to assess severity of illness. CMI is a factor for hospital reimbursement and also a method to assess average patient morbidity from year to year. CMI scoring is a relative value assigned to a DRG and is comprised of the principal and secondary diagnoses, procedures, age, and comorbidities, as well as hospital resources [21].

Primary and secondary outcomes were identified in advance of data collection and were chosen based on our institution’s existing measures for assessing the impact of stewardship interventions as well as IDSA guidance [20]. The primary outcome, median days of therapy, is a standardized method to classify antibiotic days based on patient-level exposure and is inclusive of all antibiotics prescribed for a patient throughout hospitalization [22, 23]. DOT was chosen to measure antibiotic exposure as it accounts for both dosing and frequency of each drug [23]. For example, a patient receiving pipericillin/tazobactam every 6 hours for 2.5 days and vancomycin every 12 hours for 3 days will have a DOT of 5.5 (Table 3). Secondary outcomes included hospital mortality, 30-day readmission, CDI during hospitalization, and antimicrobial ADEs during hospitalization. ADEs from antimicrobials were defined as infusion-related injury or irritation, nausea, vomiting, diarrhea, Q-T interval prolongation, or arthralgia.

Descriptive statistics were calculated to describe continuous and categorical variables including age, gender, CMI, and DRG. The Wilcoxon rank sum test was used to evaluate the statistical significance of DOT differences. The chi-square test was applied to evaluate differences in binary secondary outcomes between pre- and post-PCT-A groups: hospital mortality, 30-day readmission, CDI during hospitalization, and antimicrobial ADEs during hospitalization. Statistical analysis was completed using R version 3.1.2 statistical software.

## RESULTS

A total of 985 patient records from January 2006 to December 2009 were evaluated and compared with 1167 patients from January 2011 to December 2014, after the PCT-A was implemented. Cohort characteristics were similar among the 2152 patients included for analysis (Table 1).

**Table 1.** Cohort Characteristics of Patients in the Pre-PCT Implementation and Post-PCT Implementation Groups

Characteristic	Pre-PCT (2006**–**2009) (n = 985)	Post-PCT (2011**–**2014) (n = 1167)	*P* Value
Age, median (IQR), y	72 (61–83)	73 (62–83)	.25
Male gender, %	42.4	43.6	.61
Case mix index, mean	1.026	1.032	.06
Discharge diagnosis, n (%)			
** **Pneumonia	589 (59.8)	641 (54.9)	.02
** **COPD	166 (16.9)	291 (18.8)	<.001
** **Kidney and genitourinary infection	122 (12.4)	121 (10.4)	.14
** **Sepsis	13 (1.3)	90 (7.7)	<.001
** **Skin and skin structure infection	62 (6.3)	71 (6.1)	.83
Biliary tract infection	23 (2.3)	15 (1.3)	.07
** **Osteomyelitis	10 (1.0)	10 (0.9)	.70
ICU admission, n (%)	77 (7.82)	93 (7.97)	.90

Abbreviations: COPD, chronic obstructive pulmonary disease; ICU, intensive care unit; IQR, interquartile range; PCT, procalcitonin.

A 47% reduction was noted in DOT between the years 2011 and 2014 after the PCT-A was added to antimicrobial stewardship practices. Antimicrobial stewardship alone resulted in a median DOT of 17 (interquartile range, 8.5–22.5). Median DOT decreased to 9 (6.5–12) following the addition of the PCT-A (*P* < .0001).

Significant reductions in secondary outcomes were also seen between the 2 groups. Hospital mortality was reduced by 62% between the 2 groups (*P* < .001): 75 (7.6%) deaths occurred during hospitalization in the pre-PCT-A group vs 35 (2.9%) deaths in the post-PCT-A group.

 The 30-day readmission rate was reduced by 50% (*P* < .001): 204 (22.4%) patients were readmitted within 30 days in the pre-PCT-A group vs 119 (11.1%) patients in the post-PCT-A group.

**Figure 3.**
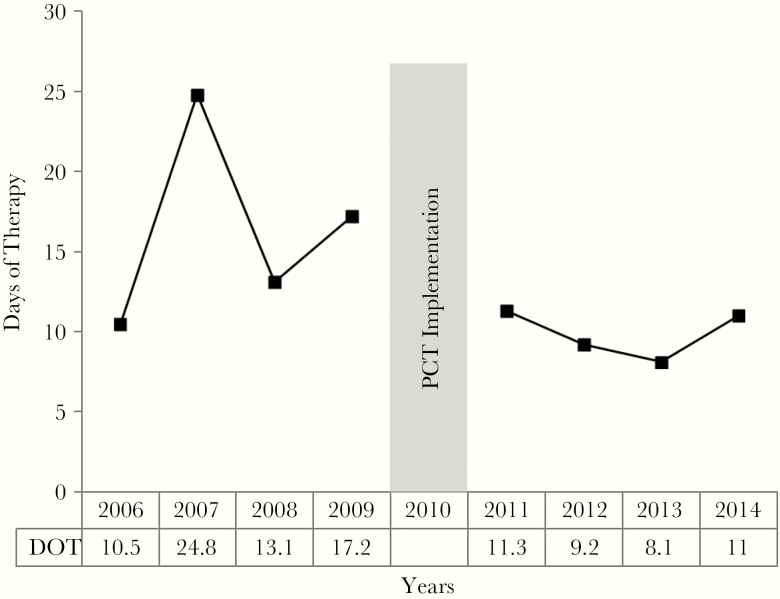
Primary outcome: days of therapy pre- and post-procalcitonin implementation. Abbreviations: DOT, days of therapy; PCT, procalcitonin.

**Figure 4.**
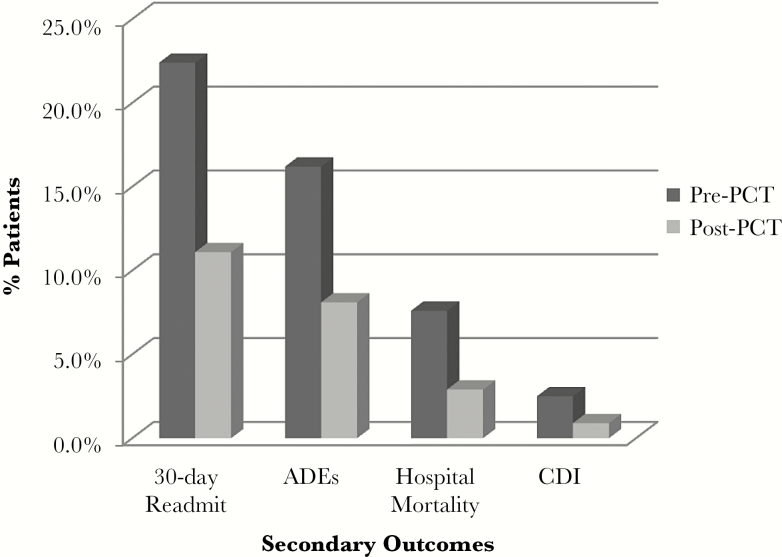
Secondary outcomes. Percentage of patients who experienced unfavorable outcomes pre-procalcitonin (PCT) compared with post-PCT-A implementation. The 30-day readmission rate was calculated by eligible readmissions (eg, # readmissions/(# patients in cohort – # in-hospital deaths)). Adverse drug events during hospitalization from antimicrobials was defined as infusion-related injury or irritation, nausea, vomiting, diarrhea, Q-T interval prolongation, or arthralgia; hospital mortality; or *Clostridium difficile* infection during hospitalization. Abbreviations: ADE, adverse drug event; CDI, *Clostridium difficile* infection; PCT, procalcitonin.

Both antimicrobial ADEs and hospital CDI rates were significantly reduced following the addition of the PCT-A. There were 160 (16.2%) ADEs in the pre-PCT-A group vs 94 (8.1%) in the post-PCT-A group (*P* < .001): a reduction of 50%. The incidence of CDI in the pre-PCT-A group was 25 (2.5%) vs 10 (0.9%) events in the post-PCT-A group, resulting in a 64% reduction (*P* = .0021). Primary and secondary outcomes are presented in Table 2 and Figures 3 and 4.

**Table 2.** Primary and Secondary Outcomes in the Pre- vs Post-PCT-A Implementation Groups

	Pre-PCT (n = 985)	Post-PCT (n = 1167)	Between- Group Difference	% Reduction	*P* Value
Primary outcome					
Days of therapy, median (IQR)	17.0 (8.5–22.5)	9.0 (6.5–12.0)	–8.0	47	<.001
Secondary outcomes					
Hospital all-cause mortality, n (%)	75 (7.6)	35 (2.9)	4.7%	62	<.001
Hospital mortality from infection, n (%)	68 (6.9)	33 (2.8)	4.1%	59	<.001
30-d all-cause readmission^a^, n (%)	204 (22.4)	119 (11.1)	11.3%	50	<.001
30-d readmission for infection^a^, n (%)	177 (19.5)	111 (9.8)	9.5%	49	<.001
Hospital *C. difficile* infection, n (%)	25 (2.5)	10 (0.9)	1.6%	64	.002
ADEs from antimicrobials^b^, n (%)	160 (16.2)	94 (8.1)	8.1%	50	<.001

Abbreviations: ADE, adverse drug event; IQR, interquartile range; PCT, procalcitonin.

^a^30-day hospital readmission rate calculated by eligible readmissions (eg, # readmissions/(# patients in cohort – # in-hospital deaths)).

^b^ADEs during hospitalization from antimicrobials defined as infusion-related injury or irritation, nausea, vomiting, diarrhea, Q-T interval prolongation, or arthralgia.

## DISCUSSION

This single-center, retrospective analysis evaluated the clinical implications of a PCT algorithm to existing antimicrobial stewardship practices. The addition of the PCT-A resulted in a significant reduction in antibiotic use, improvement in hospital mortality, 30-day readmission, CDI during hospitalization, and antimicrobial ADEs during hospitalization. Our findings demonstrated a reduction in antibiotic exposure that is consistent with findings from previous randomized controlled trials (RCTs) comparing PCT algorithms to standard of care [5–16].

Prior to the PCT-A, our institution had a mature antimicrobial stewardship program. The standard practice for evaluating suspected infection included clinical assessment, culture sensitivities, antimicrobial optimization, and traditional biomarkers such as leukocyte count, bandemia, and C-reactive protein (CRP). Leukocytes, bandemia, and fever lack specificity for bacterial infection [24]. There are multiple clinical conditions and medications that can influence these biomarkers, including corticosteroids, antirheumatic drugs, autoimmune disorders, and patients with impaired immunity [25–28]. PCT is both sensitive and specific to changes in bacterial burden [17], and it enabled clinicians to evaluate antimicrobial therapy decisions earlier and with more precision. Further, PCT has an elimination half-life of approximately 24 hours. Thus, following the initiation of appropriate antibiotic therapy, one should expect PCT to reduce by approximately 50% in daily intervals [29]. Lack of daily PCT reduction suggests that the bacterial source is not controlled [30], affording an opportunity to reassess therapy choices. Pairing clinical assessment with trends in PCT enabled our clinicians to evaluate the adequacy of antibiotic therapy, subsequent changes in treatment, and the most appropriate time for cessation on an individual patient level. This approach led to significant reductions in antibiotic exposure, hospital mortality, 30-day readmission, CDI during hospitalization, and antimicrobial ADEs during hospitalization.

**Table 3.** Example of Days of Therapy Calculation Per Patient

Drug	Dose/Frequency	Day 1	Day 2	Day 3	DOT
Piperacillin/tazobactum	4.5 mg every 6 h	4 doses	4 doses	2 doses	2.5
Vancomycin	1500 mg every 12 h	2 doses	2 doses	2 doses	3
Total DOT					5.5

Abbreviation: DOT, days of therapy.

Patient DOT was tallied for each cohort and divided by the total number of patients in each cohort (pre-PCT-A = 985, post-PCT-A = 1167). Antibiotic use and duration of therapy were tabulated to an increment of a quarter of a day.

Until recently, noninferiority studies evaluating PCT-guided antibiotic management demonstrated a reduction in antibiotic consumption without increasing adverse outcomes. The Stop Antibiotics on Procalcitonin Guidance Study (SAPS) was conducted in 15 intensive care units in the Netherlands, a country with comparatively low use of antibiotics [15]. SAPS showed a significant reduction in antibiotic exposure in the PCT algorithm arm and also noted significant reduction in 28-day and 1-year mortality. The mortality findings in SAPS, taken together with the hospital mortality reduction seen in our study, may further support the hypothesis that PCT may provide both a timely and precise assessment of individual patient response to therapy and afford opportunities for modification.

The aforementioned studies evaluated similar PCT algorithms and have repeatedly shown a safe decrease in antibiotic exposure when comparing PCT with standard of care [5–16]. However, a few studies have not come to the same conclusion [31–34]. PCT algorithms that had been repeatedly proven as safe and efficacious were modified in the aforementioned studies, and this may account for the null effect. The primary differences included cutoff values, the number and timing of measurements, and/or the use of PCT to escalate vs stop antibiotics.

We attribute the reduction in antibiotic exposure and adverse outcomes shown in our study to our decision to couple clinical judgment with PCT algorithms that had been previously validated. There were key differences in our approach that may explain the adverse outcome findings in our study. We did not limit the use of PCT-A to guide antibiotic management in lower respiratory tract infection and sepsis; rather all patients presenting with suspicion of infection were eligible for protocol inclusion. Also, the pharmacy held numerous educational events for staff. Our multifaceted approach included presentations at clinical staff meetings, written communication regarding PCT practices, algorithm pocket cards for prescribers, and review of patient cases. Additionally, PCT-A was embedded in laboratory results and was easily accessible by hovering over the result. Most importantly, educational and patient case review opportunities provided by the pharmacy prior to and throughout implementation led to a consensus in PCT practices among our providers and pharmacists. Considering the size of our facility, achieving agreement among our relatively small medical staff was attainable. Consensus among disciplines and consistency in our practices may have improved patient management and thus outcomes. Further, our stewardship process ensured that baseline PCT measurements were obtained and subsequently followed by the pharmacy at appropriate intervals in patients receiving antibiotics. The process also enabled pharmacists to adjust therapeutic regimens at the time of result, which prevented delay in intervention due to obtaining physician approval. Difficult cases were discussed with the physician and care team; however, many interventions were protocol based and acted upon immediately. In 2011, we implemented an electronic system that provided acceptance rates of pharmacist interventions. During the post-PCT-A time period (2011–2014), pharmacists’ recommendation for antibiotic initiation, revision, and cessation was accepted at a rate of 83% in 2011, which increased to 95% in 2014. Further, daily rounds included a review of all antibiotics as well as culture and PCT results. Applying the same concepts with similar agreement among disciplines could lead to comparable results in other facilities.

Although we are encouraged by the findings in our study, there are limitations. First, due to the retrospective study design and lack of randomization, it is difficult to establish PCT-A as the sole determinate of our findings. That said, potential confounders that may have influenced outcomes such as modification of clinical practice guidelines and infection control surveillance were unchanged throughout study. Second, we conducted various training sessions to introduce PCT-A and to establish consistent follow-up procedures. In doing so, the identification and management of infection may have been indirectly impacted. Third, the study design did not enable the assessment of all outcomes after discharge. Instead, the outcome measures were confined to within-hospital assessment, making findings susceptible to changes in hospital length of stay (LOS). As such, the distribution and median LOS were evaluated to assess the impact on our findings. Although median hospital LOS was the same in both cohorts (3 days), the distribution showed that early discharges (<48 hours) were observed in the post-PCT-A group (supplementary data). This finding may generate a bias toward lower in-hospital counts (deaths, ADEs) in the post-PCT-A group. Fourth, our study collected data at 1 facility; therefore, prescription bias may impact the transferability of results. Fifth, protocol adherence was not captured in typical patient charting, making it difficult to assess the protocol deviations for each patient over the 4-year implementation. Following study data collection, a documentation system that tracked pharmacy interventions was implemented. We queried 200 charts in the post-PCT-A group and found adherence to be 92% over a 2-year period. Sixth, during the study period, there was a change in our practice provider model. Prior to 2012, our medical staff was comprised of 20% PCPs and 80% hospitalists. Starting in 2012, the hospitalist service took responsibility for all inpatient management. Our medical staff consists of approximately 10 providers at a given time, and thus PCPs would have only accounted for 2 out of 10 physicians. Additionally, hospital practice guidelines did not change throughout the study period, and both PCPs and hospitalists received the same PCT education. Nevertheless, we cannot overlook the possibility that a change in medical management may have also influenced our findings. Seventh, we did not evaluate the impact of our clinical outcomes on hospital costs. Hospital cost data are under review and will be submitted for publication in the near future.

## CONCLUSION

Significant reduction in antibiotic exposure was achieved in an institution with an established stewardship program. PCT has the ability to quantify the severity of bacterial infection at the time of measurement, and serial measurements show trends in PCT production and elimination, which serve as indicators of source control. In addition to clinical judgment, a simple PCT algorithm along with dedicated oversight and thorough clinician education made improved antibiotic management and outcomes achievable. Considering the pressing need to balance early and effective antibiotics with reducing unnecessary or extended courses, every hospital, regardless of size, should continuously evaluate the impact of their antimicrobial stewardship program.

## Supplementary Data

Supplementary materials are available at *Open Forum Infectious Diseases* online. Consisting of data provided by the authors to benefit the reader, the posted materials are not copyedited and are the sole responsibility of the authors, so questions or comments should be addressed to the corresponding author.

## Supplementary Material

ofx213_supplement-data
