# Supplementary material for: Impact of Procalcitonin-Guided Antibiotic Management on Antibiotic Exposure and Outcomes: Real-world Evidence
Source: Open Forum Infect Dis. 2017 Oct 3;4(4):ofx213. doi: 10.1093/ofid/ofx213 (PMC5695623; doi:10.1093/ofid/ofx213)
Supplement: ofx213_supplement-data [file ofx213_supplement-data.pdf]

Supplemental Data

i. Diagnostic Related Group (DRG) considered for study inclusion and analysis
   1. Pneumonia (177-179, 193-195)
   2. COPD (190-192)
   3. Kidney and Genitourinary Infection (688-690)
   4. Sepsis (871-872)
   5. Biliary Tract Infection (444-446)
   6. Skin and Skin Structure Infection (572, 575, 602, 603, 622)

ii. Distribution of Infection-Related Deaths

DRG	DRG Description	Pre-PCT N=985	Post-PCT N=1167
871, 872	Sepsis	8 (0.8%)	8 (0.7%)
190, 191, 192	COPD	7 (0.7%)	9 (0.8%)
177, 178, 179 193, 194, 195	Pneumonias / Respiratory	53 (5.3%)	16 (1.4%)
	Total	68 (6.9%)	33 (2.9%)

Supplement Table 1. Infection-related deaths by DRG (Broad Category)

iii. Length of Stay (LOS) Distribution by Cohort

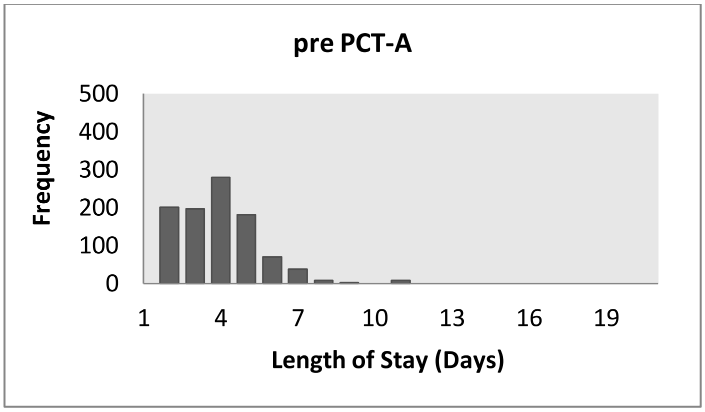

Supplement Figure 1a: Distribution of Length of Stay (LOS) pre PCT-A implementation

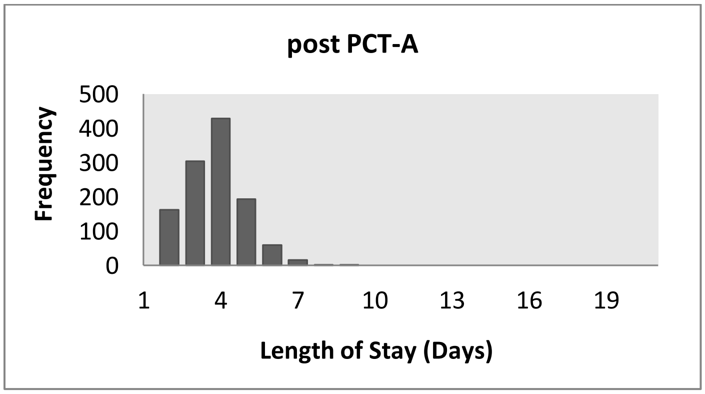

Supplement Figure 1b: Distribution of Length of Stay (LOS) post PCT-A implementation
